# Clinical simulation in the teaching of cardiovascular physical examination with nursing students: a systematic review

**DOI:** 10.1590/0034-7167-2023-0429

**Published:** 2025-03-10

**Authors:** Italo Rigoberto Cavalcante Andrade, Johny Carlos de Queiroz, Maria Sinara Farias, Nicole Paulo da Silva Maia, Lúcia de Fátima da Silva

**Affiliations:** I Universidade Estadual do Ceará. Fortaleza, Ceará, Brazil; II Universidade do Estado do Rio Grande do Norte. Mossoró, Rio Grande do Norte, Brazil; III Universidade Estadual Vale do Acaraú. Sobral, Ceará, Brazil

**Keywords:** Learning, Physical Examination, Simulation Exercise, Health Assessment, Cardiovascular Nursing, Aprendizaje, Examen Físico, Ejercicio de Simulación, Evaluación de la Salud, Enfermería Cardiovascular

## Abstract

**Objectives:**

to highlight the effect of using clinical simulation in the training of undergraduate nursing students for the development of skills in cardiovascular health assessment.

**Methods:**

a systematic review was conducted in the PubMed/MEDLINE, LILACS, Scopus, EBSCO, Web of Science, and Science Direct databases from August to September 2023. Of the 685 articles identified, 6 were selected, consisting of 4 experimental studies and 2 quasi-experimental studies, all of good methodological quality according to the Joanna Briggs Institute.

**Results:**

clinical simulation increased nursing students’ levels of knowledge and confidence. Most studies indicated that simulated practices improve learning compared to traditional methodologies.

**Conclusions:**

clinical simulation requires more robust methodological designs, and more multicenter clinical trials on cardiovascular assessment in nursing need to be published.

## RESUMEN

**Objetivos:**

evidenciar el efecto del uso de la simulación clínica en la enseñanza de estudiantes de pregrado en enfermería para el desarrollo de habilidades en la evaluación de la salud cardiovascular.

**Métodos:**

revisión sistemática realizada en las bases de datos PubMed/ MEDLINE, LILACS, Scopus, EBSCO, *Web of Science* y *Science Direct*, de agosto a septiembre de 2023. De los 685 artículos identificados, se seleccionaron 6, siendo 4 estudios experimentales y 2 cuasi-experimentales, todos con buena calidad metodológica según el Instituto Joanna Briggs.

**Resultados:**

la simulación clínica aumentó los niveles de conocimiento y confianza de los estudiantes de enfermería. La mayoría de los estudios indicaron que las prácticas simuladas mejoran el aprendizaje en comparación con las metodologías tradicionales.

**Conclusiones:**

la simulación clínica requiere diseños metodológicos más robustos, y es necesaria la publicación de más ensayos clínicos multicéntricos sobre la evaluación cardiovascular en enfermería.

## INTRODUCTION

Brazil is immersed in a national and global context of profound social, economic, and technological transformations that will have a decisive impact on the healthcare sector^(1)^. The ongoing discussions regarding the need for change and the adoption of new practices in traditional higher education stem, in large part, from the social transformations and technological advances made in recent decades, especially in health-related undergraduate programs^(2,3)^.

The academic training of nurses presents challenges in both teaching and learning, becoming a crucial process given the realities imposed by the job market. In this context, the use of active learning methodologies during university training has emerged as essential. These experiences allow students to expand their classroom environment, giving them a concrete understanding of the health system and its complexities. Over time, these experiences enable students to not only gain confidence in their ability to perform tasks but also develop autonomy in their daily professional practice^(4)^.

Among the various active methodologies described in the literature, clinical simulation stands out as a pedagogical strategy capable of creating an environment that allows students to experience real-life events through simulation, engage in clinical reasoning, and anticipate the realities of professional practice. This approach helps develop the competencies of future nurses and adds value to the learning process of nursing students^(5,6,7)^.

The primary concern in clinical simulation settings is ensuring that students can reproduce aspects of reality interactively and dynamically and, before reflecting on problem-solving in care, base their actions on scientific evidence, so that simulation has a positive impact on clinical practice^(8,9,10)^.

Given this reality within the teaching-learning process, it becomes important to emphasize the evaluation of the clinical competencies developed by nursing students, who have the opportunity to apply clinical reasoning alongside their technical skills.

Researchers have reported that students face significant challenges when performing physical examinations. The clinical techniques that posed the most difficulty in learning were auscultation and percussion^(11)^. Consequently, improving technical skills for data collection and performing nursing interventions is a critical need for undergraduate students^(12)^.

It is crucial that the knowledge addressed in undergraduate education, particularly in the teaching-learning process of cardiovascular physical examinations with nursing students, acts as a predominant factor, encompassing perceptual-cognitive, instrumental, interpersonal, and affective competencies, as well as time management skills. Additionally, it must promote changes in critical thinking, the perception of priority nursing interventions, and the many responsibilities that patient care demands.

The cardiovascular physical examination is per formed meticulously through the skills of inspection, palpation, and auscultation^(12,13)^. Professional development is crucial for improving the practice of this examination in nursing, which encourages reflection on the approach to physical examination and strengthens the quality of care provided. As a healthcare professional closely involved with the patient, the nurse plays an important role in identifying the needs of the patient, family, and community. Additionally, it is essential to promote intervention research to deepen the technical knowledge of physical examination in nursing^(14)^.

Nurses are capable of conducting an accurate clinical evaluation, which improves patient prognosis, enhances quality of life, minimizes complications, and promotes health education practices^(15)^. It is important to note that skill development should begin during undergraduate studies^(16)^. Therefore, academic institutions in the healthcare field must develop and pursue new mechanisms and strategies to ensure that the professional training of graduates aligns with new educational frameworks, meets the demands of modern society, and complies with the healthcare system, thereby ensuring quality care for the population^(17)^.

Given the prominence and growing applicability of clinical simulation in the teaching of cardiovascular physical examination, it is relevant to explore studies on this subject, justifying the need for this review.

## OBJECTIVES

To highlight the effect of using clinical simulation in the training of undergraduate nursing students for the development of skills in cardiovascular health assessment.

## METHODS

### Ethical Aspects

Since this is a systematic review that uses public domain data and does not involve human subjects, approval by the Research Ethics Committee was waived.

### Study Design

This is a systematic literature review, structured according to the Preferred Reporting Items for Systematic Reviews and Meta-Analyses (PRISMA)^(18)^. The review followed seven steps: defining the research question; identifying the databases, descriptors, keywords, and search strategies; establishing inclusion and exclusion criteria; conducting database searches; comparing the search results from the reviewers and determining the initial selection of studies; critically analyzing all studies included in the review; and preparing a critical summary^(19)^.

### Defining the Research Question

To define the research question, the Patient, Intervention, Comparison, Outcomes (PICO)^(20)^ strategy was used, with the following elements: P referred to nursing students; I to clinical simulation; C to traditional teaching and learning strategies; and O to satisfactory cardiovascular physical examination in nursing. Thus, the following question was formulated: *What is the effect of teaching through clinical simulation on the development of skills for cardiovascular health assessment in the teaching-learning process of nursing students?*

### Identifying Relevant Studies

The selected databases were PubMed/MEDLINE, *Literatura Latino-Americana e do Caribe em Ciências da Saúde* (LILACS), Scopus, EBSCO, Web of Science, and Science Direct. Based on this selection, controlled descriptors and Boolean operators (AND for the simultaneous occurrence of topics, OR for the occurrence of one or another topic) were adopted. For term selection, the descriptors found in the *Descritores em Ciências da Saúde* (DeCS) were used.

The integrated search was conducted using the following descriptors: *“Students, Nursing”, “Simulation Training”, “Physical Examination”, “Learning”.* The search strategies are presented in Chart 1. It is worth noting that in the PubMed and Scopus databases, the descriptors found in *Medical Subject Headings* (MeSH) were used.

**Chart 1:** Search strategies used in the selected databases for research, Fortaleza, Ceará, Brazil, 2023

Data base	Search strategies
PubMed/ MEDLINE	(“Students, Nursing” OR “Pupil Nurses” OR “Student, Nursing” OR “Nurses, Pupil” OR “Nurse, Pupil” OR “Pupil Nurse” OR “Nursing Student” OR “Nursing Students”) AND (“Simulation Training” OR “Training, Simulation”) OR “Interactive Learning” OR “Learning, Interactive”) AND (“Teaching” OR “Training Techniques” OR “Training Technique” OR “Technique, Training” OR “Techniques, Training” OR “Training Technics” OR “Technic, Training” OR “Technics, Training” OR “Training Technic” OR “Teaching Methods“ OR “Teaching Method” OR “Method, Teaching” OR “Methods, Teaching” OR “Academic Training” OR “Training, Academic” OR “Training Activities” OR “Training Activity” OR “Activities, Training” OR “Activity, Training” OR “Techniques, Educational” OR “Educational Techniques” OR “Educational Technique” OR “Technique, Educational” OR “Educational Technics” OR “Educational Technic” OR “Technic, Educational” OR “Technics, Educational”) AND (“Physical Examination” OR “Examinations, Physical” OR “Physical Examinations” OR “Physical Exam” OR “Exam, Physical” OR “Exams, Physical” OR “Physical Exams” OR “Examination“).
Scopus	(“Students, Nursing” AND “Simulation Training” AND “Teaching” AND “Physical Examination”); (“Students, Nursing” AND “Simulation Training” AND “Learning”); (“Students, Nursing” AND “Clinical Simulation” AND “Physical examination” AND “Cardiac”).
EBSCO	(“Students” AND “Nursing” AND “Simulation Training” AND “Physical Examination” AND “Methods”); (“Nursing Students” AND “Simulation Clinical” AND “Cardiac”); (“Students, Nursing” AND “Clinical Simulation” AND “Physical Examination” AND “Interactive Learning”).
Web of Science	(“Students, Nursing” AND “Simulation Training” AND “Teaching” AND “Physical Examination”); (“Students, Nursing” AND “Simulation Training” AND “Physical Examination”); (“Students, Nursing” AND “Simulation Training” AND “Learning” AND “Methods”); (“Nursing” AND “Clinical Simulation” AND “Exam); (“Nursing Students” AND “Exam Physical”, “Simulation”); (“Nursing Students” AND “Simulation Clinical” AND “Cardiac”).
Science Direct	(“Students” AND “Nursing” AND “Simulation Training” AND “Physical Examination” AND “Methods”); (“Students, Nursing” AND “Interactive Learning” AND “Physical Exam”); (“Students, Nursing” AND “Simulation Training” AND “Learning AND “Exams, Physical”); (“Nursing Students” AND “Simulation Clinical” AND “Physical exam” AND “Cardiac”).

### Inclusion Criteria

The inclusion criteria consisted of randomized or non-randomized clinical trials and quasi-experimental studies that demonstrated a comparison between the effect of undergraduate nursing education through clinical simulation for the development of cardiovascular assessment skills and other teaching-learning strategies. There were no time limitations, and publications in Portuguese, English, and Spanish were sought in scientific journals available in full text electronically. Studies that addressed the use of clinical simulation in continuing education for nurses, literature reviews, editorials, reviews, experience reports, case studies, theoretical reflections, dissertations, theses, monographs, and abstracts published in conference proceedings were excluded.

The studies were selected by two blind reviewers (NM and LS). Initially, titles and abstracts were reviewed (Phase 1). For studies considered eligible for inclusion, a full-text reading was carried out blindly by the same reviewers (Phase 2). In case of disagreement, a third reviewer (JF) was consulted to make the final decision. Subsequently, a critical review of the full articles was conducted.

### Study Selection

The study selection process took place from August to September 2023. Searches were conducted in selected databases and virtual libraries for the screening of titles, abstracts, and full-text readings, with the assistance of free web-based review software, *Rayyan Qatar Computing Research Institute* ^(21)^, accessible at https://rayyan.qcri.org/. The search results were exported to *Rayyan^®^ – Intelligent Systematic Review*, where duplicates were identified and excluded.

During data collection, a specific framework was used to extract information related to the research question and objectives of the review, such as authorship, year/country, objective, method, results, conclusion, and level of evidence. The Rating System for the Hierarchy of Evidence for Intervention/Treatment Questions^(22)^ was also employed, classifying the levels of evidence as follows:

- I: Evidence from systematic reviews or meta-analyses of randomized clinical trials;
- II: Evidence from randomized clinical trials;
- III: Evidence from non-randomized clinical trials;
- IV: Evidence from case-control and cohort studies;
- V: Evidence from systematic reviews of descriptive and qualitative studies;
- VI: Evidence from descriptive or qualitative studies;
- VII: Evidence from expert opinions or specialist reports.

The study selection followed the PRISMA^(18)^ guidelines, and the results were presented through a narrative synthesis, with a descriptive analysis of the data.

### Data Analysis

To critically assess the methodological quality of the selected studies, the *Joanna Briggs Institute* (JBI) tool was used, which includes nine methodological assessment items for quasi-experimental studies and 13 for experimental studies, considering whether the items are present, absent, clear, or not applicable^(23)^.

Regarding the risk of bias, the following classification was adopted:

- Low risk of bias (more than 70% of responses marked “yes”);
- Moderate risk of bias (“yes” in 50 to 69% of responses);
- High risk of bias (less than 49% of responses marked “yes”)^(24)^.

## RESULTS

**Figure 1:**
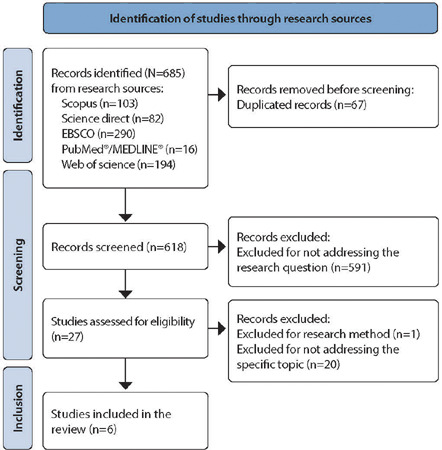
Flowchart of study selection, created based on the Preferred Reporting Items for Systematic Reviews and Meta-Analyses recommendations, Fortaleza, Ceará, Brazil, 2023

Initially, 685 studies were found, distributed across the data sources as follows: 15.1% in Scopus (103), 11.9% in Science Direct (82), 42.3% in EBSCO (290), 2.3% in PubMed/MEDLINE (16), and 28.3% in Web of Science (194). A total of 67 duplicate articles were excluded, along with 591 after reviewing titles and abstracts, leaving 27 for full-text review. Subsequently, 21 articles were excluded for not meeting the inclusion criteria of the study, leaving 6 to compose the systematic review. Figure 1 illustrates the selection and inclusion of studies in this research, in accordance with PRISMA recommendations^(18)^.

Two experimental studies^(25,26)^ and four quasi-experimental studies^(12,27,28,29)^ were included in the sample of this review. The investigations were conducted in four countries (three in North America, one in South America, and two in Asia), with sample sizes ranging from 17 to 84 participants, all of whom were nursing students. Most of the studies were published in the last 11 years. All were published in English in international journals, with one of the journals being Brazilian^(12)^.

Most of the studies contributed to the growing body of evidence that simulated practice undoubtedly impacts learning outcomes in nursing education, in addition to supporting the development of clinical skills in cardiovascular assessment. Furthermore, the authors pointed out that clinical simulation was an efficient teaching-learning method for developing clinical competencies in nursing students for the evaluation of cardiovascular physical examinations, compared to other active methodologies.

The quasi-experimental studies included were considered to be of satisfactory quality, as they met most of the requirements for methodological quality assessment^(23)^. However, the requirement for the use of a control group was not met in one of the studies. In the experimental studies, methodological weaknesses were observed regarding participant randomization, allocation to treatment groups based on blinding, the administration of treatments to blinded participants, and the identical delivery of the intervention across treatment groups^(23)^ (Chart 2).

**Chart 2:** Evaluation of the experimental and quasi-experimental studies included in the review, according to the methodological quality assessment tool of the Joanna Briggs Institute, Fortaleza, Ceará, Brazil, 2024

Evaluation of the quasi-experimental studies included in the review					
Questions	Fernandes et al.^(12)^	Jeffries et al.^(25)^	Tuzer et al.^(28)^	Menon et al.^(29)^	% of responses
1. Is it clear in the study what is the “cause” and what is the “effect,” meaning there is no confusion about which variable comes first?	Yes	Yes	Yes	Yes	100
2. Do the participants included in the groups have similar characteristics for comparison?	Yes	Yes	Yes	Yes	100
3. Did the participants in the groups receive similar treatments in the intervention of interest?	Yes	Yes	Yes	Yes	100
4. Was there a control group?	Yes	No	No	Yes	50
5. Were there multiple measurements of the outcome pre- and post-intervention/exposure over time?	Yes	Yes	No	No	50
6. Was the follow-up complete, and if not, were the differences between the groups adequately described and analyzed?	Yes	Yes	Yes	Yes	100
7. Were the outcomes of the participants, in any comparisons, measured in the same way?	Yes	Yes	Yes	Yes	100
8. Were the outcomes measured reliably?	Yes	No	Yes	Yes	75
9. Were appropriate statistical analyses used?	Yes	No	Yes	Yes	75
**Evaluation of the methodological quality of the experimental studies**					
**Questions**	**Tiffen *et al.* ^(27)^**	**Tawalbeh *et al.* ^(26)^**		**% of responses**	
1. Was randomization used to allocate participants to treatment groups?	Yes	Yes		100	
2. Was the researcher responsible for group allocation blinded?	No	No		0	
3. Were the treatment groups similar?	Yes	Yes		100	
4. Were participants blinded to the treatment allocation?	No	No		0	
5. Were the individuals administering the treatment blinded?	No	No		0	
6. Were the outcome assessors blinded to the treatment allocation?	No	No		0	
7. Were the treatment groups treated identically regarding the intervention of interest?	Yes	Yes		100	
8. Was follow-up completed, and if not, were differences between the groups in terms of follow-up adequately described and analyzed?	Yes	Yes		100	
9. Were participants analyzed in the groups to which they were allocated?	Yes	Yes		100	
10. Were the outcomes measured in the same way for the treatment groups?	Yes	Yes		100	
11. Were the outcomes measured reliably?	Yes	Yes		100	
12. Was appropriate statistical analysis used?	Yes	Yes		100	
13. Was the study design appropriate, and was there any deviation from the standard RCT* design in its conduct and analysis?	Yes	Yes		100	

*RCT – Randomized Controlled Trial.*

Chart 3 presents data regarding authorship, year of publication, objectives, design and sample size of the studies researched.

**Chart 3:** Characterization of the studies that comprised the sample regarding authorship, year, objectives, study design, and sample size, Fortaleza, Ceará, Brazil, 2024

Articles	Authorship, year/country	Objectives	Study design	Sample size
**A1** ^(12)^	Fernandes et al., 2020, Brazil.	To evaluate the effect of clinical simulation as an educational strategy with nursing students for learning about cardiovascular physical examination.	Quasi-experimental study. Intervention group (IG) – consisted of clinical simulation; Control group (CG) – received only traditional lecture. Use of the UTREND instrument (Transparent Reporting of Evaluations with Nonrandomized Designs). Simulated scenario: constructed using an adapted version of the National League for Nursing (NLN)/Jeffries Simulation model..	30 nursing students (IG – 15; CG – 15)
**A2** ^(27)^	Tiffen et al., 2011, United States.	To determine whether the use of an intermediate fidelity mannequin simulator can affect students’ confidence, knowledge, and satisfaction with physical assessment skills.	Randomized clinical trial. Initially, an in-person lecture on the cardiovascular and respiratory systems was given, followed by a practical lab session for both groups. IG – experienced an intermediate fidelity simulator (for 1 hour); CG – the usual teaching and learning strategy group did not receive any additional instruction. A cardiopulmonary skills and confidence assessment tool was administered to both groups.	28 nursing students (IG – 14; CG – 14)
**A3** ^(28)^	Tuzer et al., 2016, Turkey.	To compare the effects of using a high-fidelity simulator and standardized patients on students’ knowledge and skills in performing chest, pulmonary, and cardiac examinations, and to explore students’ views and learning experiences.	Mixed method. Students were randomly assigned to Group I – practiced chest, lung, and cardiac examinations using a high-fidelity simulator, and Group II – practiced physical examinations on standardized patients.	52 nursing students (IG – 26; GII – 26)
**A4** ^(25)^	Jeffries et al., 2011, United States.	To develop, implement, and evaluate the outcomes of a cardiovascular assessment curriculum for advanced practice nurses across four institutions.	Multicenter, prospective, quasi-experimental intervention study. Each institution used a one-group pre-post intervention design. The educational interventions included simulation-based case presentations conducted by faculty, using the Harvey® cardiopulmonary patient simulator, and independent learning sessions with the simulator itself through a CD-ROM program (Essential Cardiac Auscultation) – multimedia.	36 nursing students
**A5** ^(26)^	Tawalbeh et al., 2016, Jordan.	To test the effect of simulation on the confidence of undergraduate nursing students in applying physical examination skills for the heart and lungs.	Randomized clinical trial. IG – participated in a cardiopulmonary physical examination simulation session and a 1-hour PowerPoint and video presentation; CG – attended the same video presentation and a traditional laboratory training session. A high-fidelity simulator was used, including normal and abnormal respiratory sounds, palpable pulse, blood pressure, chest expansion, electrocardiogram monitoring, and the simulator’s voice.	84 nursing students (IG – 42; CG – 42)
**A6** ^(29)^	Menon et al., 2022, United States.	To examine the effects of using high-fidelity simulation in nursing education by comparing the performance of heart and lung physical assessment skills.	Quasi-experimental study. After randomization, all students participated in traditional didactic instruction. An application was used for the IG (with the aid of headphones) that allows the user to overlay animated 3D models of human organs on mannequins (high-fidelity simulation). The organs visualized were those relevant to cardiopulmonary assessment. CG – practiced on the mannequin without the aid of high-fidelity simulation.	17 nursing students (IG – 10; CG – 07)

*IG - Intervention Group; CG - Control Group; UTREND - Transparent Reporting of Evaluations with Nonrandomized Designs; NLN - National League for Nursing.*

Chart 4 shows the data regarding the main results, conclusion and level of evidence of the studies referenced in the systematic review.

**Chart 4:** Characterization of the studies that comprised the sample regarding main results, conclusion, and level of evidence, Fortaleza, Ceará, Brazil, 2024

Articles	Main Results	Conclusion	Level of Evidence
**A1** ^(12)^	The study demonstrated an improvement in learning when the overall performance of the IG, subjected to simulation, was compared to the CG. In the pre-test, the correct responses to each of the questions were homogeneous between the groups, with no statistical significance. In the post-test, the IG, which underwent clinical simulation, did not show statistical significance when the instrument’s questions were analyzed individually. The within-group analysis of the intervention group was conducted by comparing the mean correct responses of the intervention group at the pre-test and post-test stages. The intervention group had a median (p = 0.141) of 6.0 in the pre-test and a median (p = 0.192) of 8.0 in the post-test. After applying the Student’s t-test, a statistically significant difference was found between the means (p = 0.001), indicating an improvement in the intervention group.	The IG, subjected to clinical simulation on cardiovascular physical examination, showed improvement in overall performance. Clinical simulation had a positive effect on the learning of undergraduate nursing students.	Level 5
**A2** ^(27)^	Students in the IG acquired greater knowledge in cardiopulmonary assessment compared to the control group in the post-test. However, there was no difference in confidence between the two groups. Students with fewer years of experience showed lower levels of confidence during the assessment. The reliability of the measure was 0.81 in the pre-test and 0.80 in the post-test. Differences in the distribution of demographic data were analyzed using non-parametric chi-square tests. Differences in mean confidence, change in confidence from pre-test to post-test, and knowledge level in the post-test were analyzed using parametric t-tests. A Hotelling’s test was used to conduct multivariate analysis of each question in the confidence survey.	The study contributed to the growing body of evidence suggesting that patient simulation positively impacts learning outcomes in advanced practice nursing education, in addition to high satisfaction with the simulator used.	Level 2
**A3** ^(28)^	Knowledge and performance scores for all students increased after the simulation activities; however, students who worked with standardized patients achieved significantly higher knowledge scores than those who worked with the high-fidelity simulator. Nonetheless, there was no significant difference in performance scores between the groups. The average performance scores of students working with real patients were significantly higher compared to the post-simulation assessment scores (p < 0.001). The pre-test and post-test scores of the patients, as well as the performance scores, were converted into percentage values, and the Shapiro-Wilk test was used to assess their compliance with a normal distribution.	The results revealed that the use of standardized patients was more effective than the use of a high-fidelity simulator in increasing students’ knowledge of chest, lung, and cardiac assessments. However, practice with real patients increased the performance scores of all students, without any significant differences.	Level 5
**A4** ^(25)^	The outcome measures included a 31-item cognitive written exam, a 13-item skills checklist used in each of the three-station objective structured clinical examinations, a self-efficacy and student satisfaction survey, an instructor satisfaction and self-efficacy survey, and a participant logbook to record practice time using the self-learning materials. Thirty-six students who received simulation-based training showed a statistically significant improvement from pre-test to post-test in cognitive knowledge and cardiovascular assessment skills.	The use of the deliberate practice model and a simulation-based curriculum model to learn cardiovascular assessment and diagnostic reasoning skills was very important in this study. Students acquired essential cardiovascular clinical skills in a non-threatening, interactive, and individualized learning environment. Overall, the results support the benefit of incorporating deliberate practice using the Harvey curriculum model into a nursing program when cardiovascular knowledge and skills are required for advanced nursing practice roles.	Level 5
**A5** ^(26)^	The confidence of the students improved significantly more in the experimental group than in the control group in the first post-test. Additionally, the findings of this study supported the outcome of a significant improvement in knowledge, cardiac examination skills, identification of important auscultatory findings, and the ability to make correct diagnoses. The most important difference in the education received by the experimental group compared to the control group was the introduction of a practical and realistic environment. This environment used monitors and a simulated patient on which the students applied physical examination skills and auscultated the heart and chest for normal and abnormal respiratory sounds. The control group participants, who received only traditional training, did not perform the technical skills available to the simulation group participants. An independent t-test was used to test whether there was any statistically significant difference between the participants in the experimental and control groups in the pre-test. Additionally, the chi-square test was used to assess the presence of statistically significant differences between the study groups in terms of sex. The Cronbach’s alpha reliability was 0.85, indicating good internal consistency among the high-fidelity simulator items.	Both simulation and traditional laboratory training significantly improved participants’ confidence in applying cardiopulmonary assessment skills. However, simulation training had a more significant effect than the usual training in increasing nursing students’ confidence in applying physical examination skills.	Level 2
**A6** ^(29)^	The use of high-fidelity simulation demonstrated significant improvement in the IG for auscultation of the broncho-vesicular sounds and the aortic, pulmonary, tricuspid, and mitral valves. The areas of improvement were those reinforced by the high-fidelity simulation, such as the correct positioning for auscultation. Using this type of simulation for cardiac and pulmonary structures, along with auditory instructions, initially improved the students’ performance. Significant improvements were also observed in the CG regarding the aforementioned parameters after the study. No statistical or clinical differences were observed between the groups, although the IG performed marginally better. During the period following the initial training, the CG was able to develop a similar level of skill as the IG. Regarding student satisfaction, the simulation and self-confidence in learning were tested for reliability using Cronbach’s alpha (satisfaction = 0.94; self-confidence = 0.87). To determine the statistical significance of the use of high-fidelity clinical simulation, two-tailed unpaired t-tests were conducted on the control and experimental group datasets. The two-tailed Wilcoxon non-parametric test was also performed, resulting in no changes in the statistical significance of the parameters. Cohen’s d was used to determine the effect size in these datasets.	The results of this pilot study supported the value of this technology for nursing students. The authors will use larger sample sizes for the next study. This study suggests that high-fidelity clinical simulation is a valuable teaching tool with applications in various areas of nursing.	Level 5

*IG - Intervention Group; CG - Control Group.*

Regarding the methodological quality analyzed^(23)^, all studies presented an overall quality score above 70%, indicating good internal validity and a low risk of bias. Although the studies showed heterogeneity in their methods, as well as variations in sample size and participants’ age range, homogeneity was observed regarding the impact on nursing students’ knowledge levels, confidence, and performance improvement.

### Characterization of the research subjects

In terms of the sample characterization of the articles, A1 presented a mean age of 24.69 years for the IG and 25.76 for the CG, with a predominance of females (73%). A2 presented a mean age of 27.5 years for the IG and 28.4 for the CG, with the majority being female (92.8%). In A3, the study presented a mean age of 23 years, with 88.5% of participants being female. A4 presented an age range between 26 and 35 years (44%), without specifying the gender of the participants. The participants in A5 had a mean age of 19.1 years, with a gender distribution of males (48.80%) and females (51.20%). The article A6 did not specify the gender or age of the participants. Regarding the period of students’ education, A3 conducted the study in the fourth year of the course, and A4 in the first year.

### Student Confidence and Performance

For the experimental studies^(26,27)^, in A2, IG students showed greater knowledge during cardiac assessment compared to CG students; however, IG students did not demonstrate higher confidence compared to CG students.

In A5, confidence in applying physical examination skills was significantly higher in the post-test compared to the pre-test for both groups. An independent t-test revealed a statistically significant difference (t(67) = -42.95, p < 0.001) in terms of confidence in applying physical examination skills in the first post-test, following the intervention, between the experimental group (mean = 24.00, Standard Deviation (SD) = 1.89) and the control group (mean = 8.02, SD = 1.43). Additionally, an independent t-test indicated a statistically significant difference (t(67) = -43.36, p < 0.001) between the experimental group (mean = 25.10, SD = 1.88) and the control group (mean = 9.30, SD = 1.22) in terms of confidence in applying physical examination skills in the second post-test, administered three months after the intervention. However, it is worth noting that the IG (simulation) had a more significant effect on student confidence compared to conventional education.

For the quasi-experimental studies^(12,27,28,29)^, in A1, it was observed that, in terms of confidence, clinical simulation in cardiovascular physical examination led to an improvement in student performance compared to CG, presenting moderate evidence (Student’s t-test p = 0.05).

In A3, the performance scores obtained in the IG evaluation were significantly higher compared to those obtained in the post-test for both groups. It is worth noting that there was no significant difference in the average increase in performance scores between the IG and CG groups. Regarding skill acquisition, students in both groups achieved similar performance scores in the post-simulation evaluation test (t = 0.767, p = 0.447).

A4 reported that student confidence increased with cardiovascular assessment practice, as did their clinical reasoning skills. After correctly identifying heart sounds and findings in most cases, students’ self-confidence increased with simulation practice (on a five-point scale, the average scores ranged from 4.6 to 5.0 / 5 = strongly agree), thereby enhancing their knowledge and skills in this area. In this study, instructors reported high satisfaction and confidence in teaching cardiovascular assessment techniques using clinical simulation.

Finally, A6 addressed student satisfaction with the simulation and self-confidence in learning, yielding reliability values tested using Cronbach’s alpha (satisfaction = 0.94; self-confidence = 0.87).

## DISCUSSION

The studies compared the effect of clinical simulation on the teaching of cardiovascular assessment in nursing using medium-fidelity mannequins, simulators, and real patients, alongside traditional teaching and learning strategies, such as lectures, skills training in laboratories, and the use of low-fidelity mannequins. The majority of the studies^(12,26,27,29)^ presented statistically significant results in favor of clinical simulation for cardiovascular assessment, compared to traditional teaching methods. For the majority, aspects of satisfaction, knowledge, and self-confidence were taken into consideration.

This finding is reiterated in a randomized experimental study conducted with 76 Jordanian nursing students, which tested the effect of simulation on knowledge, confidence, and critical care skills for patients with cardiac, respiratory, and neurological problems. The t-test indicated that the mean knowledge and confidence regarding the implementation of intensive care skills were significantly higher (p < 0.001) in the post-test compared to the pre-test, in both the experimental and control groups. However, the independent t-test revealed that students in the experimental group scored significantly higher (p < 0.001) than the control group in terms of knowledge and confidence related to intensive care skills performance^(26)^. Other studies have identified clinical simulation as a transformative methodology for developing nursing students’ and nurses’ self-confidence, knowledge, and satisfaction competencies^(30,31)^.

In line with this review, another randomized study compared the effectiveness of simulation in developing nursing competencies with traditional teaching strategies, highlighting statistically significant results for the improvement of cognitive and psychomotor skills (p = 0.001) and an increase in confidence (p = 0.026)^(32)^. Another study also emphasized clinical simulation as an innovative pedagogical strategy for developing cognitive, psychomotor, and affective skills in nursing, confirming its effectiveness in fostering clinical competency growth^(33)^.

From undergraduate courses onward, the knowledge of techniques and the competencies needed to perform nursing procedures are taught. For several years now, universities have implemented new active teaching strategies, such as clinical simulation, which help develop clinical reasoning and technical skills training, contributing to both patient safety and the preparation of future professionals^(34)^.

Among the clinical skills taught in academia, physical examination is included, which is directly associated with performance that can promote patient safety and positive outcomes in clinical conditions^(35)^. However, physical examination must be dynamic, integrated, and performed meticulously, grounded in humanized care that is patient-centered and guided by clinical techniques^(16)^.

From this perspective, clinical simulation employed in undergraduate nursing education has a positive impact on the development of competencies, as well as on the increase in satisfaction, confidence, and knowledge in cardiovascular practices. Therefore, given the importance of training excellent healthcare professionals, it is crucial that this group develop skills in a safe and effective manner, especially when the focus is on nursing care in cardiovascular assessment.

For the execution of a physical examination, cardiac auscultation stands out as a fundamental clinical method that nurses must perform in a timely manner to detect early abnormal cardiac findings and recognize the patient’s clinical deteriora-tion^(36)^. In light of the responsibility to provide excellent care, nursing students need to experience scenarios that replicate real clinical conditions, so their practice is reinforced by self-confidence and self-knowledge.

Regarding knowledge of cardiopulmonary physical examination, from a theoretical perspective, there is a significant gap. Among the techniques available for cardiovascular system examination, a study conducted in Brazil found a 51.4% error rate during cardiac auscultation. The complexity associated with the multiple intricacies of correctly executing the technique represents a challenge for nursing students^(16)^.

Simulation-based education has increasingly played an important role in healthcare education worldwide, as it not only protects the patient from potential risks but is also valued for its ability to create conditions that optimize learning. Topics considered difficult to teach or understand can be selectively practiced and systematically reproduced, allowing nursing students to achieve competence through deliberate and repeated practice^(37)^.

Additionally, valuing subjective, affective, relational, and attitudinal components, along with technical-scientific aspects during the examination, can provide the nurse with a holistic approach, enabling the recognition of dimensions of the health-disease-care process that other professionals may not perceive. This contributes to consolidating a distinct field of practice for the nurse, while prioritizing the care of the patient, with information that helps improve their health condition^(38)^.

Regarding the use of the JBI instrument, there is a recognized need to implement blinding in educational experiments, especially due to the difficulty of ensuring the absence of information exchange between the participants involved^(23,37,39)^.

Based on the results of the selected studies, it can be inferred that the importance of evaluating the skills and competencies applied in the teaching-learning process in cardiovascular nursing should be extended to different educational contexts, whether in undergraduate education or in professional nursing practice.

This study has implications for reflections on the practices carried out from academic training, as well as on the deliberate experiences of nursing professionals, potentially encouraging the creation of conditions for innovative educational practice.

Considering the importance of developing competencies and skills in cardiovascular assessment in nursing through clinical simulation for undergraduate nursing students, attention is drawn to the progression in exploring and developing critical thinking in nursing students, as a controlled and safe environment fosters greater confidence, satisfaction, and knowledge among peers.

### Study limitations

This study, like any other, is not without limitations. Difficulties in comparing the results of the studies included in the sample were noted due to the use of different assessment instruments. Additionally, of the six selected articles, four conducted research with samples from a single institution.

### Contributions to the Nursing Field

This work contributes to encouraging and expanding research aimed at building scientific evidence on clinical simulation in the development of cardiovascular assessment skills in nursing, helping to improve the teaching-learning process, particularly during academic training.

## CONCLUSIONS

In this review, it became evident that clinical simulation demonstrated consistency in improving cardiovascular physical examination assessments, such as the enhancement of cognitive and psychomotor skills, increased confidence, and knowledge among undergraduate nursing students, compared to other traditional pedagogical teaching strategies.

We conclude that research in this area requires greater use of more robust methodological designs, which will enable more relevant results. Clinical simulation aims to surpass traditional strategies for skill and competency development by exploring a dynamic process that involves the creation of hypothetical, yet genuinely realistic situations. This facilitates the complex integration of practical learning with essential opportunities for repetition, helping to prevent harm to patients.
