# Supplementary material for: Clinical simulation in the teaching of cardiovascular physical examination with nursing students: a systematic review
Source: Rev Bras Enferm. 2025 Mar 10;78(1):e20230429. doi: 10.1590/0034-7167-2023-0429 (PMC11893062; doi:10.1590/0034-7167-2023-0429)
Supplement: Translation

# Translation: Simulação clínica no ensino do exame físico cardiovascular com estudantes de enfermagem: revisão sistemática

**DOI:** 10.1590/0034-7167-2023-0429pt

**Authors:** Italo Rigoberto Cavalcante Andrade, Johny Carlos de Queiroz, Maria Sinara Farias, Nicole Paulo da Silva Maia, Lúcia de Fátima da Silva

**Affiliations:** I Universidade Estadual do Ceará. Fortaleza, Ceará, Brasil; II Universidade do Estado do Rio Grande do Norte. Mossoró, Rio Grande do Norte, Brasil; III Universidade Estadual Vale do Acaraú. Sobral, Ceará, Brasil

**Keywords:** Aprendizagem, Exame Físico, Exercício de Simulação, Avaliação em Saúde, Enfermagem Cardiovascular

## Abstract

**Objetivos:**

evidenciar o efeito da utilização da simulação clínica no ensino de estudantes de graduação em enfermagem para o desenvolvimento de habilidades na avaliação em saúde cardiovascular.

**Métodos:**

revisão sistemática realizada nas bases PubMed/MEDLINE, LILACS, Scopus, EBSCO, *Web of Science* e *Science Direct*, de agosto a setembro de 2023. Dos 685 artigos identificados, 6 foram selecionados, sendo 4 estudos experimentais e 2 quase-experimentais, todos de boa qualidade metodológica, conforme o Instituto Joanna Briggs.

**Resultados:**

a simulação clínica aumentou os níveis de conhecimento e confiança dos estudantes de enfermagem. A maioria dos estudos indicou que as práticas simuladas melhoram a aprendizagem em comparação com metodologias tradicionais.

**Conclusões:**

a simulação clínica necessita de desenhos metodológicos mais robustos, sendo necessária a publicação de mais ensaios clínicos multicêntricos sobre avaliação cardiovascular em enfermagem.

## INTRODUÇÃO

O Brasil está inserido em um contexto nacional e global de profundas transformações sociais, econômicas e tecnológicas, que terão impacto decisivo na área da saúde^(^1^)^. As constantes discussões sobre as necessidades de mudanças e a adoção de novas condutas relacionadas à educação tradicional no ensino superior advêm, em grande parte, das transformações sociais e dos avanços tecnológicos desenvolvidos nas últimas décadas, especialmente nos cursos de graduação em saúde^(^2^,^3^)^.

A formação acadêmica do enfermeiro apresenta desafios no ensino e na aprendizagem, tornando-se um processo crucial diante da realidade imposta pelo mercado de trabalho. Nesse contexto, emerge a necessidade da utilização de metodologias ativas de aprendizagem durante a formação universitária. Essas vivências possibilitam ampliar o ambiente da sala de aula, permitindo ao aluno uma visão concreta do sistema de saúde e sua complexidade, viabilizando, ao longo da formação, não apenas segurança para executar o trabalho, mas também autonomia para exercer a prática profissional diária^(^4^)^.

Dentre as diversas metodologias ativas descritas na literatura, destaca-se a simulação clínica, uma estratégia pedagógica capaz de criar um ambiente que permite, por meio de representação, a vivência de um evento real, o raciocínio clínico e a antecipação da realidade da prática profissional, desenvolvendo as competências do futuro enfermeiro e agregando valor ao aprendizado do estudante de enfermagem^(^5^,^6^,^7^)^.

A principal preocupação nos cenários de simulação clínica é garantir que o aluno reproduza aspectos da realidade de forma interativa e dinâmica e, antes de refletir sobre as soluções para os problemas de cuidados, baseando-se em evidências científicas, de modo que a simulação promova um impacto positivo na prática clínica^(^8^,^9^,^10^)^.

Diante dessa realidade no processo de ensino-aprendiza-gem, torna-se importante enfatizar a avaliação da competência clínica desenvolvida pelo estudante de enfermagem, que tem a oportunidade de aplicar o raciocínio clínico, bem como suas habilidades técnicas.

Pesquisadores relataram que os estudantes apresentam maior dificuldade na realização do exame físico. As técnicas propedêuticas que demandaram mais dificuldade no aprendizado foram a ausculta e a percussão^(^11^)^. Consequentemente, aperfeiçoar as habilidades técnicas para a obtenção de dados e a realização de intervenções de enfermagem constitui uma necessidade para os estudantes de graduação^(^12^)^.

É indispensável que o conhecimento abordado na graduação, no processo de ensino-aprendizagem do exame físico cardiovascular com estudantes de enfermagem, funcione como fator preponderante, envolvendo competências perceptivo-cognitivas, instrumentais, interpessoais, afetivas, de gestão dos recursos no tempo, além de promover mudanças no pensamento crítico, percepção da intervenção de enfermagem prioritária e das inúmeras responsabilidades que o cuidado ao paciente exige.

O exame físico cardiovascular é realizado de forma criteriosa por meio das habilidades de inspeção, palpação e ausculta^(^12^,^13^)^. O aperfeiçoamento profissional é crucial para aprimorar a prática desse exame na enfermagem, o que contribui para uma reflexão sobre a abordagem do exame físico e fortalece a qualidade da assistência prestada. O enfermeiro, como profissional próximo ao paciente, desempenha um papel importante na identificação das necessidades do paciente, da família e da comunidade. Além disso, é fundamental estimular pesquisas de intervenção para aprofundar o conhecimento técnico do exame físico na enfermagem^(^14^)^. O enfermeiro é capaz de realizar uma avaliação clínica precisa, o que melhora o prognóstico do paciente, além de proporcionar uma melhor qualidade de vida, minimizar complicações e promover práticas de educação em saúde^(^15^)^. Ressalta-se que o aprimoramento de habilidades deve ocorrer ainda na graduação^(^16^)^.

Assim, é necessário que as instituições acadêmicas da área da saúde criem e busquem novos mecanismos e estratégias para que a formação profissional dos egressos ocorra de acordo com as novas configurações de ensino, atendendo aos anseios da sociedade moderna e estando em conformidade com o sistema de saúde, garantindo a qualidade assistencial à população^(^17^)^. Diante da notoriedade e do crescimento da aplicabilidade da simulação clínica no ensino do exame físico cardiovascular, torna-se pertinente buscar conhecimentos nos estudos sobre a temática em questão, justificando a realização desta revisão.

## OBJETIVOS

Evidenciar o efeito da utilização da simulação clínica no ensino de estudantes de graduação em enfermagem para o desenvolvimento de habilidades na avaliação em saúde cardiovascular

## MÉTODOS

### Aspectos éticos

Por se tratar de uma revisão sistemática que utiliza dados de domínio público e não envolve seres humanos, a apreciação pelo Comitê de Ética em Pesquisa foi dispensada.

### Desenho do estudo

Trata-se de uma revisão sistemática da literatura, estruturada por meio da estratégia *Preferred Reporting Items for Systematic Reviews and Meta-Analyses (PRISMA)* ^(^18^)^. Para sua elaboração, seguiram-se sete etapas: definição da pergunta de pesquisa; identificação das bases de dados, descritores, palavras-chave e estratégias de busca; estabelecimento de critérios de inclusão e exclusão; busca nas bases de dados; comparação das buscas dos examinadores e definição da seleção inicial dos estudos; análise crítica de todos os estudos incluídos na revisão; e elaboração de um resumo crítico^(^19^)^.

### Identificação da questão de pesquisa

Para a definição da questão de pesquisa, foi utilizada a estratégia *Patient, Intervention, Comparison, Outcomes* (PICO)^(^20^)^, com os seguintes elementos: o acrônimo P se referiu aos estudantes de enfermagem; I, à simulação clínica; C, às estratégias tradicionais de ensino e aprendizagem; e O, ao exame físico cardiovascular satisfatório em enfermagem. Dessa forma, estruturou-se a seguinte questão: *Qual é o efeito do ensino por simulação clínica no desenvolvimento de habilidades para a avaliação em saúde cardiovascular no processo de ensino-aprendizagem de estudantes de enfermagem?*

### Identificação dos estudos relevantes

As bases de dados selecionadas foram: PubMed/MEDLINE, Literatura Latino-Americana e do Caribe em Ciências da Saúde (*LILACS*), Scopus, EBSCO, *Web of Science* e *Science Direct*. A partir dessa seleção, adotaram-se descritores controlados e operadores booleanos AND, para a ocorrência simultânea de assuntos, e OR, para a ocorrência de um ou outro assunto. Para a escolha dos termos, foram utilizados os descritores encontrados nos *Descritores em Ciências da Saúde (DeCS)*.

A busca integrada foi realizada com os seguintes descritores: *“Students, Nursing”, “Simulation Training”, “Physical Examination”, “Learning”*. As estratégias de busca estão apresentadas no Quadro 1. Destaca-se que, nas bases de dados PubMed e Scopus, foram utilizados os descritores encontrados no *Medical Subject Headings (MeSH)*.

**Quadro 1:** Estratégias de busca utilizadas nas bases de dados selecionadas para pesquisa, Fortaleza, Ceará, Brasil, 2023

Bases de dados	Estratégias de busca
PubMed/ MEDLINE	*(“Students, Nursing” OR “Pupil Nurses” OR “Student, Nursing” OR “Nurses, Pupil” OR “Nurse, Pupil” OR “Pupil Nurse” OR “Nursing Student” OR “Nursing Students”) AND (“Simulation Training” OR “Training, Simulation”) OR “Interactive Learning” OR “Learning, Interactive”) AND (“Teaching” OR “Training Techniques” OR “Training Technique” OR “Technique, Training” OR “Techniques, Training” OR “Training Technics” OR “Technic, Training” OR “Technics, Training” OR “Training Technic” OR “Teaching Methods“ OR “Teaching Method” OR “Method, Teaching” OR “Methods, Teaching” OR “Academic Training” OR “Training, Academic” OR “Training Activities” OR “Training Activity” OR “Activities, Training” OR “Activity, Training” OR “Techniques, Educational” OR “Educational Techniques” OR “Educational Technique” OR “Technique, Educational” OR “Educational Technics” OR “Educational Technic” OR “Technic, Educational” OR “Technics, Educational”) AND (“Physical Examination” OR “Examinations, Physical” OR “Physical Examinations” OR “Physical Exam” OR “Exam, Physical” OR “Exams, Physical” OR “Physical Exams” OR “Examination“).*
Scopus	*(“Students, Nursing” AND “Simulation Training” AND “Teaching” AND “Physical Examination”); (“Students, Nursing” AND “Simulation Training” AND “Learning”); (“Students, Nursing” AND “Clinical Simulation” AND “Physical examination” AND “Cardiac”).*
EBSCO	*(“Students” AND “Nursing” AND “Simulation Training” AND “Physical Examination” AND “Methods”); (“Nursing Students” AND “Simulation Clinical” AND “Cardiac”); (“Students, Nursing” AND “Clinical Simulation” AND “Physical Examination” AND “Interactive Learning”).*
Web of Science	*(“Students, Nursing” AND “Simulation Training” AND “Teaching” AND “Physical Examination”); (“Students, Nursing” AND “Simulation Training” AND “Physical Examination”); (“Students, Nursing” AND “Simulation Training” AND “Learning” AND “Methods”); (“Nursing” AND “Clinical Simulation” AND “Exam); (“Nursing Students” AND “Exam Physical”, “Simulation”); (“Nursing Students” AND “Simulation Clinical” AND “Cardiac”).*
Science Direct	*(“Students” AND “Nursing” AND “Simulation Training” AND “Physical Examination” AND “Methods”); (“Students, Nursing” AND “Interactive Learning” AND “Physical Exam”); (“Students, Nursing” AND “Simulation Training” AND “Learning AND “Exams, Physical”); (“Nursing Students” AND “Simulation Clinical” AND “Physical exam” AND “Cardiac”).*

Para os critérios de inclusão, elegeram-se ensaios clínicos, aleatorizados ou não, e estudos quase-experimentais, capazes de demonstrar a comparação entre o efeito do ensino de graduação em enfermagem, por meio da simulação clínica, para desenvolver habilidades na avaliação cardiovascular com outras estratégias de ensino-aprendizagem. Não houve delimitação temporal, e foram buscadas publicações nos idiomas português, inglês e espanhol, em periódicos científicos disponíveis eletronicamente na íntegra. Excluíram-se estudos que abordassem o uso de simulação clínica na educação permanente de enfermeiros, revisões de literatura, editoriais, resenhas, relatos de experiências, estudos de caso, reflexões teóricas, dissertações, teses, monografias e resumos publicados em anais de eventos.

Os estudos foram selecionados por dois revisores cegos (NM e LS). Inicialmente, foram revisados títulos e resumos (Fase 1). Nos casos em que os estudos foram considerados elegíveis para inclusão, uma leitura do texto completo foi realizada às cegas pelos mesmos revisores (Fase 2). Em caso de divergência, um terceiro revisor (JF) foi consultado para tomar a decisão final. Posteriormente, foi realizada uma investigação crítica dos artigos na íntegra.

### Seleção dos estudos

A etapa de seleção dos estudos ocorreu no período de agosto a setembro de 2023. Realizou-se a busca em bases de dados e bibliotecas virtuais selecionadas para triagem de títulos, resumos e leitura de textos completos, com o auxílio de um programa de revisão gratuito da web, de versão única, chamado *Rayyan Qatar Computing Research Institute* ^(^21^)^, acessível no link https://rayyan.qcri.org/. Os resultados da pesquisa foram exportados para o software *Rayyan^®^ – Intelligent Systematic Review*, no qual duplicatas foram identificadas e excluídas.

Na coleta dos dados, utilizou-se um roteiro específico, extraindo informações relacionadas à questão e ao objetivo da revisão, como: autoria, ano/país, objetivo, método, resultados, conclusão e nível de evidência. Optou-se também por utilizar o *Rating System for the Hierarchy of Evidence for Intervention/Treatment Questions* ^(^22^)^, que classifica os níveis de evidência da seguinte forma:

- I: Evidências de revisões sistemáticas ou metanálise de ensaios clínicos randomizados;
- II: Evidências de ensaios clínicos randomizados;
- III: Evidências de ensaios clínicos sem randomização;
- IV: Evidências de estudos caso-controle e de coorte;
- V: Evidências de revisões sistemáticas de estudos descritivos e qualitativos;
- VI: Evidências de estudos descritivos ou qualitativos;
- VII: Evidências de opinião de autoridades ou relatórios de especialistas.

A seleção dos estudos seguiu as recomendações *PRISMA* ^(^18^)^ e a apresentação dos resultados foi realizada por meio de síntese narrativa, com análise descritiva dos dados.

### Análise dos dados

Para a obtenção de um cenário de avaliação crítica da qualidade metodológica dos estudos selecionados, optou-se por utilizar o instrumento do *Joanna Briggs Institute (JBI)*, que possui nove itens de avaliação metodológica voltados para estudos quase-experi-mentais e 13 para experimentais, considerando se os itens estão presentes, ausentes, se há clareza ou se não se aplicam^(^23^)^.

No que se refere ao risco de viés, adotou-se a seguinte classificação:

- Baixo risco de viés (mais de 70% das respostas assinaladas como “sim”);
- Risco moderado de viés (“sim” em 50 a 69% das respostas);
- Alto risco de viés (menos de 49% das respostas assinaladas como “sim”)^(^24^)^.

## RESULTADOS

Inicialmente, foram encontrados 685 estudos, distribuídos entre as fontes de dados da seguinte forma: 15,1% na Scopus (103), 11,9% na *Science Direct* (82), 42,3% na EBSCO (290), 2,3% na PubMed/MEDLINE (16) e 28,3% na *Web of Science* (194). Foram excluídos 67 artigos duplicados e 591 após a leitura de títulos e resumos, restando 27 para leitura na íntegra. Subsequentemente, 21 artigos foram excluídos por não atenderem aos critérios de inclusão da pesquisa, restando 6 para compor a revisão sistemática. A Figura 1 ilustra a seleção e a inclusão dos estudos da presente pesquisa, conforme as recomendações PRISMA^(^18^)^.

**Figura 1:** Fluxograma de seleção dos estudos, elaborado a partir da recomendação *Preferred Reporting Items for Systematic Reviews and Meta-Analyses*, Fortaleza, Ceará, Brasil, 2023

Dois estudos experimentais^(^25^,^26^)^ e quatro quase-experimen-tais^(^12^,^27^,^28^,^29^)^ foram incluídos na amostra desta revisão. As investigações foram conduzidas em 4 países (3 na América do Norte, 1 na América do Sul e 2 na Ásia), com tamanho amostral variando entre 17 e 84 participantes, todos estudantes de enfermagem. A maioria dos estudos foi publicada nos últimos 11 anos. Todos foram publicados em inglês, em periódicos internacionais; uma das revistas é brasileira^(^12^)^.

A maior parte dos estudos contribuiu para o crescente corpo de evidências de que a prática simulada impacta, indubitavelmente, os resultados de aprendizagem na educação em enfermagem, além de contribuir para o desenvolvimento de habilidades clínicas na avaliação cardiovascular. Não obstante, os autores apontaram que a simulação clínica foi um processo de ensino-aprendizagem eficiente para desenvolver competências clínicas em estudantes de enfermagem para a avaliação do exame físico cardiovascular, quando comparada a outras metodologias ativas.

Os estudos quase-experimentais incluídos foram considerados de qualidade satisfatória, uma vez que atenderam à maioria das exigências para avaliação de qualidade metodológica^(^23^)^. Apenas o quesito que exige o uso de grupo controle não foi atendido em um dos estudos. Contudo, nos estudos exper imentais, obser vou-se fragilidade metodo -lógica quanto à aleatorização dos participantes, à alocação nos grupos de tratamento conforme cegamento, à condução do tratamento em participantes cegados, e à intervenção de interesse de forma idêntica entre os grupos de tratamento^(^23^)^ (Quadro 2).

**Quadro 2:** Avaliação dos estudos experimentais e quase-experimentais incluídos na revisão, de acordo com o instrumento de avaliação de qualidade metodológica do Instituto Joanna Briggs, Fortaleza, Ceará, Brasil, 2024

Avaliação dos estudos quase-experimentais incluídos na revisão					
Perguntas	Fernandes et al.^(^12^)^	Jeffries et al.^(^25^)^	Tuzer et al.^(^28^)^	Menon et al.^(^29^)^	% de respostas
1. Está claro no estudo o que é a “causa” e o que é o “efeito”, ou seja, não há confusão sobre qual variável vem primeiro?	Sim	Sim	Sim	Sim	100
2. Os participantes incluídos nos grupos têm características semelhantes para comparação?	Sim	Sim	Sim	Sim	100
3. Os participantes incluídos nos grupos receberam tratamento semelhantes na intervenção de interesse?	Sim	Sim	Sim	Sim	100
4. Houve grupo controle?	Sim	Não	Não	Sim	50
5. Existiram múltiplas medições do resultado pré e pós-intervenção/exposição ao longo do tempo?	Sim	Sim	Não	Não	50
6. O acompanhamento foi completo e, caso contrário, as diferenças entre os grupos foram descritas e analisadas de forma adequada?	Sim	Sim	Sim	Sim	100
7. Os resultados dos participantes, em quaisquer comparações, foram medidos da mesma maneira?	Sim	Sim	Sim	Sim	100
8. Foram medidos os resultados de maneira confiável?	Sim	Não	Sim	Sim	75
9. Foram usadas as análises estatísticas apropriadas?	Sim	Não	Sim	Sim	75
**Avaliação da qualidade metodológica dos estudos experimentais**					
**Perguntas**	**Tiffen *et al.* ^(^27^)^**	**Tawalbeh *et al.* ^(^26^)^**		**% de respostas**	
1. A aleatorização foi utilizada para alocar os participantes aos grupos de tratamento?	Sim	Sim		100	
2. O pesquisador responsável pela alocação nos grupos de tratamento estava cegado?	Não	Não		0	
3. Os grupos de tratamento foram semelhantes?	Sim	Sim		100	
4. Os participantes foram cegados na alocação do tratamento?	Não	Não		0	
5. Os responsáveis por fornecerem o tratamento estavam cegados?	Não	Não		0	
6. Os avaliadores de resultados estavam cegados quanto à alocação do tratamento?	Não	Não		0	
7. Os grupos de tratamento foram tratados de forma idêntica à intervenção de interesse?	Sim	Sim		100	
8. O acompanhamento foi concluído e, se não, as diferenças entre os grupos, em termos de acompanhamento, foram adequadamente descritas e analisadas?	Sim	Sim		100	
9. Os participantes foram analisados nos grupos para os quais foram alocados?	Sim	Sim		100	
10. Os resultados foram mensurados da mesma maneira para os grupos de tratamento?	Sim	Sim		100	
11. Os resultados foram mensurados de maneira confiável?	Sim	Sim		100	
12. Foi utilizada a análise estatística apropriada?	Sim	Sim		100	
13. O desenho do estudo foi apropriado, e houve qualquer desvio do desenho padrão do ECR* na condução e análise?	Sim	Sim		100	

*ECR – Ensaio Clínico Randomizado.*

O Quadro 3 apresenta os dados referentes a autoria, ano de publicação, objetivos, desenho e tamanho da amostra dos estudos pesquisados.

**Quadro 3:** Caracterização dos estudos que compuseram a amostra quanto à autoria, ano, objetivos, desenho do estudo e tamanho da amostra, Fortaleza, Ceará, Brasil, 2024

Artigos	Autoria, ano/país	Objetivos	Desenho do estudo	Tamanho da amostra
**A1** ^(^12^)^	Fernandes et al., 2020, Brasil.	Avaliar o efeito da simulação clínica como estratégia educativa com estudantes de enfermagem para aprendizagem sobre exame físico cardiovascular.	Estudo quase-experimental. Grupo intervenção (GI) – constou de simulação clínica; Grupo-controle (GC) – recebeu apenas a aula tradicional Uso do instrumento UTREND (*Transparent Reporting of Evaluations with Nonrandomized Designs*). Cenário simulado: construído utilizando, de forma adaptada, o modelo *National League for Nursing* (NLN)/*Jeffries Simulation.*	30 estudantes de enfermagem (GI – 15; GC – 15)
**A2** ^(^27^)^	Tiffen et al., 2011, Estados Unidos.	Determinar se o uso de um simulador de manequim de fidelidade intermediária pode afetar a confiança, o conhecimento e a satisfação do aluno com as habilidades de avaliação física.	Ensaio clínico randomizado. Inicialmente, foi ministrada uma aula presencial sobre sistemas cardiovascular e respiratório, seguido de aula prática em laboratório para os dois grupos. GI – experiência com simulador de fidelidade intermediária (por 1 hora); GC – grupo de estratégia de ensino e aprendizagem usual não recebeu nenhuma instrução adicional. Aplicado instrumento de avaliação de habilidades cardiopulmonares e confiança de ambos os grupos.	28 estudantes de enfermagem (GI – 14; GC – 14)
**A3** ^(^28^)^	Tuzer et al., 2016, Turquia.	Comparar os efeitos do uso de um simulador de alta fidelidade e pacientes padronizados sobre o conhecimento e as habilidades dos alunos na realização de exames tórax, pulmonares e cardíacos, e explorar a visão e as experiências de aprendizagem dos alunos.	Método misto. Os alunos foram atribuídos aleatoriamente ao Grupo I – praticou exame do tórax, pulmão e cardíaco usando um simulador de alta fidelidade e, ao Grupo II – praticou exame físico em pacientes padronizados.	52 estudantes de enfermagem (GI – 26; GII – 26)
**A4** ^(^25^)^	Jeffries et al., 2011, Estados Unidos.	Desenvolver, implementar e avaliar os resultados de um currículo de avaliação cardiovascular para enfermeiros de prática avançada em quatro instituições.	Estudo de Intervenção multicêntrico, prospectivo, quase experimental. Cada instituição usou um desenho pré-pós-intervenção de um grupo. As intervenções educacionais incluíram apresentações de casos baseados em simulação conduzida pelo corpo docente, usando o simulador cardiopulmonar de paciente Harvey® e sessões de aprendizagem independentes com o próprio simulador, por meio de um programa de CD-ROM (*Essential Cardiac Auscultation*) - multimídia.	36 estudantes de enfermagem.
**A5** ^(^26^)^	Tawalbeh et al., 2016, Jordânia.	Testar o efeito da simulação na confiança de estudantes universitários de enfermagem na aplicação de habilidades de exame físico do coração e pulmão.	Ensaio clínico randomizado. GI – participou de uma sessão de simulação de exame físico cardiopulmonar e uma apresentação de PowerPoint e vídeo de 1 hora; GC – assistiu à mesma apresentação de vídeo e uma sessão de treinamento laboratorial tradicional. Foi utilizado um simulador de alta fidelidade, incluindo sons respiratórios normais e anormais, pulso palpável, pressão arterial, expansão torácica, monitoramento de eletrocardiograma e voz do simulador.	84 estudantes de enfermagem (GI – 42; GC – 42)
**A6** ^(^29^)^	Menon et al., 2022, Estados Unidos.	Examinar os efeitos do uso da simulação de alta fidelidade no ensino de enfermagem, comparando o desempenho de habilidades de avaliação física de coração e pulmão.	Estudo quase-experimental. Após a randomização, todos os alunos participaram de instrução didática tradicional. Foi utilizado para o GI um aplicativo (com o auxílio de fones de ouvido) que permite ao usuário sobrepor modelos 3D de órgãos humanos animados em manequins (simulação de alta fidelidade). Os órgãos visualizados são aqueles relevantes para a avaliação cardiopulmonar. GC – realizou a prática no manequim sem o auxílio da simulação de alta fidelidade.	17 estudantes de enfermagem. (GI –10; GC – 07)

*GI – Grupo intervenção; GC – Grupo controle; UTREND - Transparent Reporting of Evaluations with Nonrandomized Designs; NLN - National League for Nursing.*

O Quadro 4 demonstra os dados referentes aos principais resultados, conclusão e nível de evidência dos estudos referenciados na revisão sistemática.

**Quadro 4:** Caracterização dos estudos que compuseram a amostra quanto aos principais resultados, conclusão e nível de evidência, Fortaleza, Ceará, Brasil, 2024

Artigos	Principais resultados	Conclusão	Nível de Evidência
**A1** ^(^12^)^	O estudo demonstrou que houve melhora no aprendizado, quando avaliado o desempenho geral do GI, submetido a simulação, em comparação ao GC. No pré-teste, os acertos de cada uma das questões entre os grupos apresentaram-se de forma homogênea, não havendo significância estatística. No pós-teste, o GI, submetido a simulação clínica, não apresentou significância estatística quando analisadas as questões do instrumento de forma individual. A análise intragrupo do grupo intervenção foi realizada a partir da verificação da média de acertos do grupo- intervenção nos momentos pré e pós-teste. O grupo-intervenção apresentou mediana (*p* = 0,141) de 6,0 no pré-teste; e mediana (*p* = 0,1 92) de 8,0 no pós-teste. Após a aplicação do teste *t* de *Student*, encontrou-se diferença estatística significante entre as médias (*p* = 0,001), indicando a melhora do grupo-intervenção.	O GI, submetido a simulação clínica sobre exame físico cardiovascular apresentou melhora no desempenho geral. A simulação clínica apresentou efeito positivo para a aprendizagem de estudantes de graduação em Enfermagem.	Nível 5
**A2** ^(^27^)^	Os alunos do GI adquiriram maior conhecimento na avaliação cardiopulmonar em comparação aos alunos do grupo de controle no pós-teste. No entanto, não houve diferença na confiança entre os dois grupos. Alunos com menos anos de experiência obtiveram menores níveis de confiança durante avaliação. A confiabilidade da medida foi de 0,81 no préteste e 0,80 nos pós-teste. As diferenças na distribuição dos dados demográficos foram analisadas com testes não paramétricos do qui-quadrado. As diferenças na confiança média, mudança na confiança do pré-teste para o pós-teste e o nível de conhecimento no pós-teste foram analisados por meio de parâmetros de testes *t* paramétricos. Um teste de *Hotelling* foi usado para realizar a análise multivariada de cada questão na pesquisa de confiança.	O estudo contribuiu para o crescente corpo de evidências que sugere que a simulação do paciente afeta positivamente os resultados de aprendizagem na educação de enfermagem de prática avançada, além de uma alta satisfação com o simulador utilizado.	Nível 2
**A3** ^(^28^)^	As pontuações de conhecimento e desempenho de todos os alunos aumentaram após as atividades de simulação; no entanto, os alunos que trabalharam com pacientes padronizados alcançaram escores de conhecimento significativamente mais altos do que aqueles que trabalharam com o simulador de alta fidelidade; no entanto, não houve diferença significativa nas pontuações de desempenho entre os grupos. As pontuações médias de desempenho de alunos em pacientes reais foram significativamente maiores em comparação com as pontuações de avaliação pós-simulação (p < 0,001). Os escores do pré-teste e pós-teste dos pacientes e os escores de desempenho foram convertidos em valores percentuais, e o teste de *Shapiro Wilk* foi usado para testar sua conformidade com a distribuição normal.	Os resultados revelaram que o uso de pacientes padronizados foi mais eficaz que o uso de um simulador de alta fidelidade para aumentar o conhecimento dos alunos sobre avaliação do tórax, pulmão e cardíaca. Contudo, a prática em pacientes reais aumentou as pontuações de desempenho de todos os alunos, sem qualquer diferença significativa.	Nível 5
**A4** ^(^25^)^	As medidas de resultado incluíram um exame escrito cognitivo de 31 itens, uma lista de verificação de habilidades de 13 itens usada em cada um de um exame clínico estruturado objetivo de três estações, pesquisa de autoeficácia e satisfação do aluno, pesquisa de satisfação e autoeficácia do instrutor, e um diário de bordo do participante para registrar o tempo de prática usando os materiais de autoaprendizagem. Trinta e seis alunos que receberam o treinamento baseado em simulação mostraram melhoria estatisticamente significativa do pré ao pós-teste no conhecimento cognitivo e nas habilidades de avaliação cardiovascular.	O uso do modelo de prática deliberada e um modelo de currículo baseado na simulação para aprender avaliação cardiovascular e habilidades de raciocínio diagnóstico foi muito importante neste estudo. Os alunos adquiriram importantes habilidades clínicas cardiovasculares de forma não ameaçadora, interativa e individualizada num ambiente de aprendizagem. No geral, os resultados substanciam o benefício de incorporar o uso deliberado de prática no modelo do currículo Harvey em um programa de enfermagem quando problemas cardiovasculares conhecimentos e habilidades são necessários em funções de prática de enfermagem avançada.	Nível 5
**A5** ^(^26^)^	A confiança dos acadêmicos melhorou significativamente mais no grupo experimental do que no grupo de controle no primeiro pós-teste. Além disso, os achados deste estudo apoiaram o resultado de uma melhora significativa no conhecimento, habilidades de exame cardíaco, identificação de achados auscultatórios importantes, capacidade de fazer diagnósticos corretos. A diferença mais importante na educação recebida pelo grupo experimental em comparação com o grupo de controle foi a introdução de um ambiente prático e realista. Este ambiente usou monitores e um paciente simulado no qual os alunos aplicaram as habilidades de exame físico e auscultaram o coração e o tórax em busca de sons respiratórios normais e anormais. Os participantes do grupo de controle, que receberam apenas treinamento tradicional, não realizaram as habilidades técnicas disponíveis para os participantes do grupo de simulação. Um teste *t* independente foi utilizado para testar se havia alguma diferença estatisticamente significativa entre os participantes dos grupos experimental e controle no pré-teste. Além disso, o qui-quadrado foi utilizado para avaliar a presença de diferenças estatisticamente significativas entre os grupos de estudo em termos de sexo. A confiabilidade de *alfa de Cronbach* foi de 0,85, indicando boa consistência interna entre os itens do simulador de alta fidelidade	Tanto a simulação quanto o treinamento tradicional em laboratório melhoraram significativamente a confiança dos participantes na aplicação das habilidades de avaliação cardiopulmonar. No entanto, o treinamento de simulação teve um efeito mais significativo do que o treinamento usual em aumentar a confiança dos estudantes de enfermagem na aplicação de habilidades de exame físico.	Nível 2
**A6** ^(^29^)^	O uso da simulação de alta fidelidade demonstrou melhora significativa no GI nas auscultas broncovesiculares e das valvas aórtica, pulmonar, tricúspide e mitral. As áreas de melhoria foram aquelas reforçadas pela simulação alta fidelidade, como o posicionamento correto da ausculta. Utilizar esse tipo de simulação nas estruturas cardíacas e pulmonares, juntamente com as instruções auditivas, melhorou inicialmente o desempenho dos alunos. Foram observadas também grandes melhorias no GC em relação aos parâmetros supracitados após o estudo. Não houve diferenças estatísticas ou clínicas observadas entre os grupos, embora o GI tenha tido um desempenho marginalmente melhor. Durante o período após o treinamento inicial, o GC foi capaz de desenvolver um nível de habilidade semelhante ao do GI. Em relação à satisfação dos alunos a simulação e a autoconfiança na aprendizagem foi testada a confiabilidade por meio do *alfa de Cronbach* (satisfação = 0,94; autoconfiança = 0,87). Para determinar a significância estatística do uso de simulação clínica de alta fidelidade, testes *t* bicaudais não pareados foram realizados nos conjuntos de dados do grupo controle e experimental. O teste não paramétrico de *Wilcoxon* bicaudal também foi realizado, resultando em nenhuma alteração na significância estatística dos parâmetros. O *D* de *Cohen* foi usado para determinar o tamanho do efeito nesses conjuntos de dados.	Os resultados deste estudo piloto apoiaram o valor desta tecnologia para estudantes de enfermagem. Os autores usarão tamanhos de amostra maiores para o próximo estudo. Este estudo sugere que a simulação clínica de alta fidelidade é uma valiosa ferramenta de ensino com aplicações em diversas áreas da enfermagem.	Nível 5

*GI – Grupo intervenção; GC – Grupo controle.*

Quanto à qualidade metodológica analisada^(^23^)^, todos os estudos apresentaram um escore geral de qualidade superior a 70%, indicando boa validade interna e baixo risco de viés.

Embora os estudos apresentassem heterogeneidade em seus métodos, bem como variações no tamanho da amostra e na faixa etária dos participantes, observou-se homogeneidade em relação ao impacto nos níveis de conhecimento, confiança e melhoria de desempenho dos estudantes de enfermagem.

### Caracterização dos sujeitos da pesquisa

No que se refere à caracterização amostral dos artigos, o A1 apresentou uma média de idade dos participantes de 24,69 anos para o GI e 25,76 para o GC, além de predominar o sexo feminino (73%). O A2 apresentou uma idade média de 27,5 anos no GI e 28,4 no GC, sendo a maioria do sexo feminino (92,8%). No A3, o estudo apresentou uma média de idade de 23 anos e 88,5% pertenciam ao sexo feminino. O A4 apresentou uma faixa etária entre 26 e 35 anos (44%), sem especificar o sexo dos participantes. Os participantes do A5 tinham uma idade média de 19,1 anos, com uma equivalência nos percentuais de sexo (masculino – 48,80%; feminino – 51,20%). O artigo A6 não especificou o sexo e a idade dos participantes. Em relação ao período de graduação dos estudantes, o A3 desenvolveu o estudo no quarto ano do curso, e o A4, no primeiro ano.

### Confiança e desempenho dos alunos

Para os artigos experimentais^(^26^,^27^)^, no A2, os alunos do GI apresentaram maior conhecimento durante a avaliação cardíaca em relação aos alunos do GC; porém, os alunos do GI não obtiveram maior confiança em comparação aos alunos do GC.

Quanto ao A5, a confiança na aplicação das habilidades do exame físico foi significativamente maior no pós-teste em relação ao pré-teste para ambos os grupos. Um teste t independente revelou uma diferença estatisticamente significativa (t(67) = -42,95, p < 0,001) em termos de confiança na aplicação de habilidades de exame físico no primeiro pós-teste, após a intervenção, entre o grupo experimental (média = 24,00, Desvio Padrão (DP) = 1,89) e o grupo de controle (média = 8,02, DP = 1,43). Além disso, um teste t independente indicou uma diferença estatisticamente significativa (t(67) = -43,36, p < 0,001) entre o grupo experimental (média = 25,10, DP = 1,88) e o grupo controle (média = 9,30, DP = 1,22) em termos de confiança na aplicação de habilidades de exame físico no segundo pós-teste, aplicado três meses após a intervenção. No entanto, cabe destacar que o GI (simulação) teve um efeito mais significativo na confiança dos alunos em comparação com a educação convencional.

Quanto aos estudos quase-experimentais^(^12^,^27^,^28^,^29^)^, no A1, foi observado, no quesito confiança, que a simulação clínica no exame físico cardiovascular foi responsável por uma melhora no desempenho dos alunos em comparação com o GC, apresentando evidência moderada (teste t de *Student* de p = 0,05).

No A3, as pontuações de desempenho obtidas na avaliação do GI foram significativamente maiores em comparação com as obtidas no pós-teste de ambos os grupos. Vale destacar que não houve diferença significativa no aumento médio dos escores de desempenho entre os grupos GI e GC. Quanto à aquisição de habilidades, os alunos de ambos os grupos alcançaram escores de desempenho semelhantes no teste de avaliação pós-simulação (t = 0,767, p = 0,447).

O A4 relatou que a confiança dos alunos aumentou com a prática da avaliação cardiovascular, assim como suas habilidades de raciocínio clínico. Após identificarem os sons cardíacos e os achados corretamente na maioria dos casos, a autoconfiança dos alunos aumentou com a prática da simulação (na escala de cinco itens, as pontuações médias variaram de 4,6 a 5,0 / 5 = concordo totalmente), facilitando, assim, o conhecimento e as habilidades dos alunos nessa área. Neste estudo, os instrutores relataram alta satisfação e confiança com o ensino da técnica de avaliação cardiovascular utilizando a simulação clínica.

Por fim, o A6 abordou a satisfação dos alunos com a simulação e a autoconfiança na aprendizagem, obtendo como resultado os valores de confiabilidade testados por meio do alfa de Cronbach (satisfação = 0,94; autoconfiança = 0,87).

## DISCUSSÃO

Os estudos compararam o efeito da simulação clínica no ensino da avaliação cardiovascular em enfermagem utilizando manequins de média fidelidade, simuladores e pacientes reais com estratégias de ensino e aprendizagem tradicionais, como aula expositiva, capacitação de habilidades em laboratório e o uso de manequins de baixa fidelidade. A maioria dos estudos^(^12^,^26^,^27^,^29^)^ apresentou resultados estatisticamente significativos para a simulação clínica na avaliação cardiovascular, em comparação com os métodos tradicionais de ensino. Para essa maioria, foram levados em consideração os aspectos de satisfação, conhecimento e autoconfiança.

Essa afirmação é reiterada em um estudo experimental rando-mizado, realizado com 76 estudantes jordanianos de enfermagem, que testou o efeito da simulação no conhecimento, na confiança e no desempenho de habilidades de cuidados críticos para pacientes com problemas cardíacos, respiratórios e neurológicos. O teste t indicou que o conhecimento médio e a confiança em relação à implementação de habilidades de cuidados intensivos foram significativamente maiores (p < 0,001) no pós-teste em comparação ao pré-teste, tanto no grupo experimental quanto no grupo controle. No entanto, o teste t independente revelou que os alunos do grupo experimental pontuaram significativamente mais alto (p < 0,001) do que o grupo controle em conhecimento e confiança em relação ao desempenho de habilidades de cuidados intensivos^(^26^)^. Outros estudos apontaram a simulação clínica como uma metodologia transformadora para o desenvolvimento de competências em autoconfiança, conhecimento e satisfação de estudantes de enfermagem e enfermeiros^(^30^,^31^)^.

Em consonância com esta revisão, outro estudo randomizado comparou a efetividade da simulação no desenvolvimento de competências em enfermagem com estratégias tradicionais de ensino, destacando resultados estatisticamente significativos para o aprimoramento de habilidades cognitivas e psicomotoras (p = 0,001) e a elevação da confiança (p = 0,026)^(^32^)^. Outro estudo também destacou a simulação clínica como uma estratégia pedagógica inovadora para o desenvolvimento de habilidades cognitivas, psicomotoras e afetivas em enfermagem, confirmando sua efetividade para o crescimento da competência clínica^(^33^)^.

Desde o curso de graduação, o conhecimento da técnica e as competências necessárias para executar os procedimentos de enfermagem são ensinados. Há alguns anos, universidades têm implementado novas estratégias ativas de ensino, como a simulação clínica, que auxilia no desenvolvimento do raciocínio clínico e no treinamento de habilidades técnicas, contribuindo tanto para a segurança dos pacientes quanto para a formação dos futuros profissionais^(^34^)^.

Dentre as habilidades clínicas ensinadas na academia, inclui-se o exame físico, diretamente associado a um desempenho que pode promover a segurança do paciente e bons resultados em condições clínicas^(^35^)^. Contudo, o exame físico deve ser dinâmico, integrado e realizado minuciosamente, pautando-se no cuidado humanizado, direcionado e orientado para o paciente, mediante técnicas propedêuticas^(^16^)^.

Nessa perspectiva, a simulação clínica empregada na graduação de enfermagem tem um impacto positivo no desenvolvimento de competências, assim como no aumento da satisfação, da confiança e do conhecimento em práticas de cunho cardiovascular. Portanto, diante da importância de formar profissionais de saúde de excelência, é crucial que esse público desenvolva habilidades de forma segura e eficaz, especialmente quando o foco está nos cuidados de enfermagem na avaliação cardiovascular.

Para a execução de um exame físico, destaca-se a ausculta cardíaca como um método propedêutico fundamental, que o profissional enfermeiro deve executar oportunamente para a detecção precoce de achados cardíacos anormais, bem como para o reconhecimento da piora clínica do paciente^(^36^)^. Diante da responsabilidade de promover uma assistência de excelência, o estudante de enfermagem precisa vivenciar cenários que possam reproduzir condições clínicas reais, para que sua prática se fortaleça na autoconfiança e no autoconhecimento.

Em relação ao conhecimento sobre o exame físico cardior-respiratório, do ponto de vista teórico, observa-se uma falha significativa. Dentre as técnicas disponíveis para o exame físico do sistema cardiovascular, um estudo realizado no Brasil observou 51,4% de erros durante a ausculta cardíaca. A complexidade associada às múltiplas peculiaridades da correta execução da técnica representa um desafio para os estudantes de enfermagem^(^16^)^.

O ensino baseado em simulação tem desempenhado um papel cada vez mais importante na educação em saúde em todo o mundo, pois, além de proteger o paciente de possíveis riscos, a simulação clínica é valorizada por sua capacidade de criar condições que otimizam o aprendizado. Conteúdos considerados difíceis de serem ensinados ou compreendidos podem ser seletivamente treinados e sistematicamente reproduzidos, permitindo que os estudantes de enfermagem alcancem a competência por meio de condutas deliberadas e repetidas^(^37^)^.

Além disso, valorizar, no momento do exame, os componentes subjetivos, afetivos, relacionais e atitudinais, além dos técnico-cien-tíficos, pode proporcionar ao enfermeiro uma atenção holística, permitindo-lhe perceber dimensões do processo saúde-doença--cuidado que outros profissionais podem não reconhecer. Isso contribui para consolidar um campo de atuação próprio para o enfermeiro, ao mesmo tempo que privilegia o ser cuidado, com informações que ajudam a melhorar sua condição de saúde^(^38^)^.

No que diz respeito ao uso do instrumento JBI, percebe-se a necessidade de realizar cegamentos em experimentos educacionais, principalmente devido à dificuldade de garantir a ausência de troca de informações entre os participantes envolvidos^(^23^,^37^,^39^)^.

De acordo com os resultados dos estudos selecionados, infere-se que a relevância de avaliar as habilidades e competências aplicadas no processo de ensino-aprendizagem na enfermagem cardiovascular deve ser estendida a diferentes contextos educacionais, sejam eles destacados na graduação ou na prática profissional do enfermeiro.

Este estudo traz implicações para reflexões sobre as condutas praticadas desde a formação acadêmica, bem como sobre as experiências deliberadas pelos profissionais de enfermagem, podendo instigar a criação de condições para uma prática educacional inovadora.

Levando-se em consideração a importância para o desenvolvimento de competências e habilidade na avaliação cardiovascular em enfermagem, por meio da simulação clínica para estudantes de graduação em enfermagem, atina-se a percepção da progressão em explorar e desenvolver o pensamento crítico de estudantes de enfermagem, visto que um ambiente controlado e seguro favorece maior confiança, satisfação e conhecimento entre os pares.

### Limitações do estudo

Este estudo, como qualquer outro, não está isento de limitações. Destacam-se as dificuldades para comparar os resultados dos estudos que compuseram a amostra, devido à utilização de diferentes instrumentos de avaliação. Além disso, dos seis artigos selecionados, quatro realizaram a pesquisa com amostragem em uma única instituição.

### Contribuições para a área da Enfermagem

O trabalho apresenta contribuições para fomentar e ampliar a realização de pesquisas direcionadas à construção de evidências científicas sobre a simulação clínica no desenvolvimento de habilidades para a avaliação cardiovascular em enfermagem, colaborando com o aprimoramento do processo de ensino--aprendizagem, principalmente durante a formação acadêmica.

## CONCLUSÕES

Nesta revisão, ficou evidente que a simulação clínica demonstrou homogeneidade na melhoria da avaliação do exame físico cardiovascular, como o aperfeiçoamento de habilidades cognitivas e psicomotoras, o aumento da confiança e o conhecimento de estudantes de graduação em enfermagem, quando comparada a outras estratégias pedagógicas de ensino tradicionais.

Concluímos que as pesquisas desenvolvidas nesta área de conhecimento exigem uma maior utilização de desenhos metodológicos mais robustos, que possibilitem resultados mais relevantes. A simulação clínica tem o propósito de superar estratégias tradicionais de desenvolvimento de habilidades e competências, explorando um processo dinâmico que envolve a criação de situações hipotéticas, mas genuinamente reais. Isso facilita a integração complexa do aprendizado prático com oportunidades de repetição essenciais, capazes de evitar danos ao paciente.
